# Exploring the Complexities of Quality Measurement in Assisted Living in Washington State

**DOI:** 10.1093/geroni/igaa057.277

**Published:** 2020-12-16

**Authors:** Carolyn Ham, Cathy McAvoy, Roger Gantz, Lindsay Schwartz

**Affiliations:** 1 Washington State Department of Health, Olympia, Washington, United States; 2 Washington State Department of Social and Health Services, Olympia, Washington, United States; 3 American Health Care Association/ National Center for Assisted Living, Pittsboro, North Carolina, United States

## Abstract

In Assisted Living (AL) choice and quality of life are highly valued by residents. Prior research has found many interrelated components contribute to AL residents’ experiences of quality. There are 543 licensed AL facilities in Washington state, with 33,830 licensed beds; in 2019, resident cost per year ranged from $44,000 to $78,000. In 2018, the Washington Legislature identified a need for consumer access to unbiased information on AL quality and directed the Department of Social and Health Services (DSHS) to form a workgroup on quality measures. The workgroup consists of representatives from state agencies, AL provider associations, advocacy organizations, AL providers and AL residents. To inform the workgroup, DSHS conducted a study of AL quality measures. Five statewide programs were identified and analyzed in 10 categories. The WA workgroup then assessed a series of measures across six domains: community participation and quality of life; consumer satisfaction; equity, diversity and inclusivity; informed choice and decision making; person-centered planning; and, resident safety. The workgroup assessed and chose to adopt CoreQ, a satisfaction measure developed by the American Health Care Association/National Center for Assisted Living (AHCA/NCAL) and endorsed by the National Quality Forum (NQF). In evaluating potential measures of quality, workgroup members weighed regulatory, industry, provider, consumer and family perspectives. Outcomes of this process include assessment of AL quality measurement in other states, selected measures for Washington and key insights into relative prioritization of quality domains by different stakeholder types.

